# Transforming Mental Health Delivery Through Behavioral Economics and Implementation Science: Protocol for Three Exploratory Projects

**DOI:** 10.2196/12121

**Published:** 2019-02-12

**Authors:** Rinad S Beidas, Kevin G Volpp, Alison N Buttenheim, Steven C Marcus, Mark Olfson, Melanie Pellecchia, Rebecca E Stewart, Nathaniel J Williams, Emily M Becker-Haimes, Molly Candon, Zuleyha Cidav, Jessica Fishman, Adina Lieberman, Kelly Zentgraf, David Mandell

**Affiliations:** 1 Department of Psychiatry Perelman School of Medicine University of Pennsylvania Philadelphia, PA United States; 2 Department of Medical Ethics and Health Policy Perelman School of Medicine University of Pennsylvania Philadelphia, PA United States; 3 Leonard Davis Institute of Health Economics University of Pennsylvania Philadelphia, PA United States; 4 Center for Health Incentives and Behavioral Economics Leonard Davis Institute of Health Economics University of Pennsylvania Philadelphia, PA United States; 5 Department of Medicine Perelman School of Medicine University of Pennsylvania Philadelphia, PA United States; 6 Department of Health Care Management The Wharton School University of Pennsylvania Philadelphia, PA United States; 7 Penn Medicine Center for Health Care Innovation University of Pennsylvania Philadelphia, PA United States; 8 Crescenz VA Medical Center Philadelphia, PA United States; 9 Department of Family and Community Health School of Nursing University of Pennsylvania Philadelphia, PA United States; 10 School of Social Policy and Practice University of Pennsylvania Philadelphia, PA United States; 11 Department of Psychiatry Columbia University College of Physicians and Surgeons New York, NY United States; 12 School of Social Work Boise State University Boise, ID United States; 13 Annenberg School for Communication University of Pennyslvania Philadelphia, PA United States

**Keywords:** implementation science, behavioral economics, mental health

## Abstract

**Background:**

Efficacious psychiatric treatments are not consistently deployed in community practice, and clinical outcomes are attenuated compared with those achieved in clinical trials. A major focus for mental health services research is to develop effective and cost-effective strategies that increase the use of evidence-based assessment, prevention, and treatment approaches in community settings.

**Objective:**

The goal of this program of research is to apply insights from behavioral economics and participatory design to advance the science and practice of implementing evidence-based practice (EBP) for individuals with psychiatric disorders across the life span.

**Methods:**

Project 1 (Assisting Depressed Adults in Primary care Treatment [ADAPT]) is patient-focused and leverages decision-making heuristics to compare ways to incentivize adherence to antidepressant medications in the first 6 weeks of treatment among adults newly diagnosed with depression. Project 2 (App for Strengthening Services In Specialized Therapeutic Support [ASSISTS]) is provider-focused and utilizes normative pressure and social status to increase data collection among community mental health workers treating children with autism. Project 3 (Motivating Outpatient Therapists to Implement: Valuing a Team Effort [MOTIVATE]) explores how participatory design can be used to design organizational-level implementation strategies to increase clinician use of EBPs. The projects are supported by a Methods Core that provides expertise in implementation science, behavioral economics, participatory design, measurement, and associated statistical approaches.

**Results:**

Enrollment for project ADAPT started in 2018; results are expected in 2020. Enrollment for project ASSISTS will begin in 2019; results are expected in 2021. Enrollment for project MOTIVATE started in 2018; results are expected in 2019. Data collection had begun for ADAPT and MOTIVATE when this protocol was submitted.

**Conclusions:**

This research will advance the science of implementation through efforts to improve implementation strategy design, measurement, and statistical methods. First, we will test and refine approaches to collaboratively design implementation strategies with stakeholders (eg, discrete choice experiments and innovation tournaments). Second, we will refine the measurement of mechanisms related to heuristics used in decision making. Third, we will develop new ways to test mechanisms in multilevel implementation trials. This trifecta, coupled with findings from our 3 exploratory projects, will lead to improvements in our knowledge of what causes successful implementation, what variables moderate and mediate the effects of those causal factors, and how best to leverage this knowledge to increase the quality of care for people with psychiatric disorders.

**Trial Registration:**

ClinicalTrials.gov NCT03441399; https://www.clinicaltrials.gov/ct2/show/NCT03441399 (Archived by WebCite at http://www.webcitation.org/74dRbonBD)

**International Registered Report Identifier (IRRID):**

DERR1-10.2196/12121

## Introduction

### Background

Worldwide, psychiatric disorders account for more years lived with disability than any other category of disease [1]. The risk of premature mortality of people with severe psychiatric disorders is elevated [2], and the annual burden to the US economy is approximately half a trillion dollars, less than half of which is due to the cost of treatment [3]. Efficacious treatments are not consistently deployed in community practice, and clinical outcomes are attenuated compared with those achieved in clinical trials [4-6]. A major frontier for mental health services research is to develop effective and cost-effective strategies that increase the use of evidence-based assessment, prevention, and treatment approaches in community settings [7]. Although the field of implementation science has offered many new frameworks that identify factors associated with the use of evidence-based practice (EBP) in health and mental health care [8,9], there is still much potential to be realized in developing and testing new approaches that more successfully increase the use of EBP. The goal of our Advanced Laboratories for Accelerating the Reach and Impact of Treatments for Youth and Adults with Mental Illness (ALACRITY) Center is to apply behavioral economics and participatory design to accelerate the reach and impact of treatments for individuals with psychiatric illness across the life span.

### Gaps in the Field of Implementation Science

On average, it takes 17 years for 14% of research to make its way into practice, with the majority of research findings never deployed in the community [10]. This finding has galvanized the development of implementation science, a discipline that has evolved rapidly, and focuses on the scientific study of methods to increase the adoption, implementation, and sustainment of EBP [11]. Implementation strategies are the interventions of implementation science. Early implementation research tested strategies that primarily involved training clinicians in EBP, based on assumptions that clinicians did not use them because they lacked knowledge and skills. Findings from a number of randomized controlled trials suggested that training improved clinicians’ knowledge of and attitudes toward EBP but did not lead to practice change [12-14]. This body of research highlighted contextual factors, such as clinician motivation and organizational culture, typically considered nuisance factors in efficacy trials, as important and understudied variables in their own right [15,16].

Implementation research in both health and mental health care began prioritizing the identification of determinants at clinician, organization, and system levels that affect implementation success or failure. Several heuristic implementation frameworks that attempted to capture constructs at each of these levels supported these studies [8,9,17]. Broadly, these studies used either qualitative methods to elucidate barriers to or facilitators of implementation [18-21] or quantitative methods to test associations between determinants and implementation outcomes such as fidelity to EBPs [22-26]. Furthermore, 3 major gaps have emerged from research that our center aims to address: (1) implementation science has not leveraged the rich literature from behavioral economics, (2) implementation research encourages stakeholder involvement but has not yet operationalized how best to do so, and (3) implementation research lacks causal theory.

### Implementation Science Has Not Leveraged the Rich Literature From Behavioral Economics

Implementation studies historically have been premised on the assumption that clinicians make rational clinical choices that maximize utility for themselves and their patients [27]. A growing body of research from the field of behavioral economics suggests that this is not how clinicians make decisions. Behavioral economics includes a set of models and frameworks that recognize that individuals tend to make decisions under the constraints of bounded rationality [28]. In other words, clinicians do not always make decisions based on complete information, exhaustive analysis of all potential outcomes, and maximization of expected utility. Instead, individuals are influenced by myriad psychological, social, cognitive, and emotional factors and a wide range of simplifying cognitive heuristics or shortcuts when making decisions. Clinicians likely are influenced in their decision making about which treatment to use and how to use it by heuristics such as availability bias (a case seen recently is particularly salient), hindsight bias (tendency to infer causality from a recent event even if it was not predictable), and status quo bias (the tendency to stick with the approach they usually use even if new and better approaches may now be available) [29,30].

To date, implementation research has not leveraged insights from behavioral economics to design implementation strategies. This approach is promising and has been applied outside of the scope of implementation science with regard to physician and patient behavior in health care [31-34]. The application of this approach necessarily moves the field away from implementation strategies designed to increase knowledge and toward strategies such as changing the environment (ie, choice architecture) to make it easier to do the desired thing, making EBP use the default, and using incentives and rewards to leverage cognitive heuristics. Incentives refer to informing individuals that they will receive rewards if they perform a behavior. Rewards refer to giving an individual money, vouchers, or valued objects or status when the behavior is performed [35].

### Implementation Research Encourages Stakeholder Involvement but Has Not Operationalized How to Do So

Although implementation science underscores the importance of stakeholder involvement in the implementation process [36], there has been little study on how to systematically involve stakeholders, such as patients, providers, administrators, payers, and policy makers, in the development, refinement, and testing of implementation strategies [37]. Engaging stakeholders systematically can increase the specificity, accuracy, and success of implementation strategies. Participatory design approaches, which emphasize active involvement of stakeholders in the design process, can be used to include stakeholder input in the process of designing and refining implementation strategies [38].

### Implementation Science Lacks Causal Theory

Causal theory is largely underdeveloped in implementation science [11,39], and there is a limited understanding of the mechanisms by which implementation strategies work [29,40]. One major rate-limiting step is that randomized controlled trials of implementation strategies rarely incorporate formal tests of mediating mechanisms [40]. This is due in part to the underdevelopment of rigorous statistical methods to test mediating and moderating effects of hypothesized mechanisms in a multilevel context. Implementation trials almost always are clustered, with patients nested within clinicians and clinicians within organizations [8,9,17]. Furthermore, implementation strategies can be directed at patients, clinicians, or organizations, and strategies at 1 level may target behavior and outcomes at other levels. For example, changes in organizational climate may affect clinician behavior and patient outcomes. There are few validated statistical approaches to test these pathways, sometimes referred to as *complex moderated mediation* or *conditional indirect effects* in a multilevel context, thus limiting the forward movement of the field in understanding how or when implementation strategies are most effective.

A second factor that limits the development of causal theory in implementation science is the lack of standardized and validated measures that assess putative mechanisms that link strategies to outcomes. Important work is currently underway to address this measurement gap in some areas of implementation science [41]; however, constructs from the field of behavioral economics, including measures that assess cognitive heuristics, have been notably absent. This is an important gap given the potential promise of these heuristics as a lever for behavior change.

Even when measures are available to assess putative mediating mechanisms and investigators test these variables as mediators within randomized controlled trials, a third factor that limits the development of causal theory in implementation science is the failure of many implementation strategies to engage the targeted mechanisms [40]. A recent systematic review of 88 randomized controlled implementation trials in mental health service settings found no evidence that any implementation strategy engaged its targeted mechanisms of action [40]. One potentially important reason for this is that, to date, the design of implementation strategies has not incorporated systematic end-user feedback and perspectives. Studies have shown that intervention design is significantly improved when it systematically elicits end-user feedback about behavioral bottlenecks and other barriers to enactment of the targeted behavior and incorporates this feedback into intervention design [42].

The center is funded as part of the National Institute of Mental Health ALACRITY P50 to support the rapid development, testing, and refinement of novel and integrative approaches for optimizing the effectiveness of treatments for and prevention of mental disorders and organizing and delivering mental health services in community settings [43]. The major aim of the Penn ALACRITY center is to accelerate the pace at which effective treatments for mental disorders are deployed in community practice, thereby increasing their impact on improving quality of life for people with these disorders, and to advance the science of implementation. The Penn ALACRITY center is intended to support research that demonstrates high synergy across disciplines and that increases the public health impact of existing and emerging mental health interventions and service delivery strategies. The Penn ALACRITY center addresses these goals with the following objectives:

Objective 1: Apply innovative, interdisciplinary approaches from behavioral economics to implementation science.

Objective 2: Apply methods from participatory design to ensure that the stakeholders’ voice is included in the development of implementation strategies in a systematic, rigorous, and collaborative manner.

Objective 3: Develop statistical approaches that allow for the elucidation of mechanisms and causal theory.

## Methods

### Overview

The Methods Core is the foundation of the Penn ALACRITY center. Specifically, it supports 3 incubators related to implementation strategy design, measurement, and statistical methods: (1) optimization of implementation strategy design through our design incubator, (2) refinement of measurement of mechanisms through our measurement incubator, and (3) development of novel approaches to test mechanisms in multilevel implementation trials through our statistical methods incubator. The Methods Core supports 3 exploratory projects that are wide in scope and span the most salient levels at which implementation takes place—the individual in treatment, the clinician, and the organization. Although each project stands alone, they are linked through common methods and measurement tools (see Table 1).

Project 1 (Assisting Depressed Adults in Primary care Treatment [ADAPT]) compares the effectiveness of different schedules of financial incentives to increase medication adherence among adults recently diagnosed with depression in primary care settings. Project 2 (App for Strengthening Services In Specialized Therapeutic Support [ASSISTS]) examines the effectiveness of normative pressure in increasing the use of EBP among frontline clinicians working with children with autism in schools. Project 3 (Motivating Outpatient Therapists to Implement: Valuing a Team Effort [MOTIVATE]) develops and tests the acceptability of organization-focused implementation strategies to increase clinicians’ use of EBPs in community mental health clinics. In the section that follows, we will describe the major activities of the Methods Core, followed by more in-depth descriptions of each project.

### Methods Core

#### Design Incubator: Optimize Implementation Strategy Design

The design incubator will test several participatory approaches to develop implementation strategies. Here, we describe 4 methods including innovation tournaments, behavioral design, rapid-cycle prototyping, and discrete choice experiments.

Innovation tournaments [44,45] take a collaborative and systematic approach to addressing complex and relatively unstructured problems using ideas from end users. Innovation tournaments begin when a host calls for ideas in an area of interest. End users are invited to submit ideas, which go through sequential stages of screening and evaluation by crowdsourcing peer review and expert input to filter and shape the raw ideas into the most promising ideas. At the end of the tournament, a few winning ideas are selected. Although innovation tournaments are solution-focused, they have added benefits related to team building and shifting organizational climate to be more egalitarian so that end users have direct input. Innovation tournaments have been successfully used in many contexts to increase stakeholder engagement, including quality improvement in health care [44,45], but have not been used to address challenges of implementing EBP in mental health services. In addition, 2 of our 3 projects (ASSISTS and MOTIVATE) include this approach. In ASSISTS, we use innovation tournaments to engage therapists in designing nonfinancial incentive strategies to improve the use of EBP among one-to-one aides working with school-age children with autism. Although we propose to leverage normative pressure to increase use of 1 EBP, data collection, there are many ways normative pressures can be applied, and there may be other incentives that may be equally or more effective, which we can learn about from our stakeholders. In MOTIVATE, we use innovation tournaments to engage clinicians in identifying the best way for organizations to use financial and nonfinancial incentives to help clinicians implement EBP.

Behavioral design is a systematic approach, informed by engineering and human-centered design principles, to understand human behavior and apply those insights to the design of behavior change interventions [46,47]. In this 5-step approach, designers first define the problem and then diagnose the problem from a behavioral lens, using qualitative and quantitative data about the context of the target behavior. The diagnosis process yields hypotheses informed by behavioral insights about the channels or barriers to the desired behavior. Next, these hypotheses are translated into potential solutions. Design solutions are also informed by behavioral insights. One design approach, developed by the UK Behavioural Insights Team, is the Easy, Attractive, Social, and Timely Framework, which organizes design solutions into factors that make the desired behavior *Easy, Attractive, Social, and Timely* [48]. For example, creating default solutions (which people will naturally stick with) makes a behavior very easy. Providing peer comparisons makes a behavior social, as most of us care how we do relative to peers, and what others think of us. Designed solutions are then tested and scaled through rigorous experiments. Project MOTIVATE employs behavioral design to generate solutions to improve the implementation of EBPs in community mental health settings. The contextual data for diagnosis phase will comprise, in part, the ideas submitted in the innovation tournament.

**Table 1:** Comparisons across the 3 exploratory projects.

Attributes	Project 1: ADAPT^a^	Project 2: ASSISTS^b^	Project 3: MOTIVATE^c^
Ecological level	Patient	Clinician	Organization
Population	Adults with depression	Youth with autism	A wide range of diagnoses and ages
Type of incentive	Financial	Social	Mix
Outcome	Medication adherence	Data collection	Acceptability of implementation strategies

^a^ADAPT: Assisting Depressed Adults in Primary care Treatment.

^b^ASSISTS: App for Strengthening Services In Specialized Therapeutic Support.

^c^MOTIVATE: Motivating Outpatient Therapists to Implement: Valuing a Team Effort.

Rapid-cycle prototyping is an industry innovation that has recently been applied to health care and is a complementary approach to behavioral design. The goal of rapid-cycle prototyping is to test potential innovations more efficiently, less expensively, and more reliably than traditional clinical trials [49]. These approaches leverage mini-pilots, or experiments that are integrated within operations, to learn how to best design strategies. Rapid-prototyping does not rely on a finished product to test. Rather, mock-ups or inexpensive versions are tested before completing the product. For example, when IBM wanted to test how users would respond to speech recognition software, it placed a hidden typist in another room who could hear the speaker through a microphone, rather than developing this complex technology first [50]. Rapid-cycle prototyping has been used extensively at the Penn Medicine Center for Health Care Innovation in a variety of clinical contexts as a way to quickly learn from successive iterations of a new technology. We use rapid-cycle prototyping in 1 of our projects. In ASSISTS, our digital tool to collect data and improve implementation will rely heavily on rapid-cycle prototyping to iteratively test the interface, information content, and response to a phone-based data collection app for providers of one-on-one behavioral support for children with autism.

Discrete choice experiments [51,52] are frequently applied in health economics as a way to rate the acceptability of programs. They have not been used to provide input on the design of implementation strategies although they represent another promising approach to increasing stakeholder engagement [53]. Discrete choice experiments are a technique for systematically eliciting individual preferences for options and their specific attributes. By systematically eliciting tradeoffs among constructed outcome combinations, discrete choice experiments generate data that can quantify relative utility or satisfaction for the presented option as well as its specific attributes. This strategy allows for eliciting preferences for treatments that do not currently exist or that individuals have not yet experienced. We use discrete choice experiments in MOTIVATE to evaluate the acceptability of collaboratively developed implementation strategies targeting organizations to increase clinician EBP implementation.

#### Measurement Incubator: Refine Measurement of Mechanisms Related to Clinician Factors

Implementation science frameworks posit that individual factors such as motivation, self-efficacy, knowledge, and attitudes are important in the implementation process [8,9]. To date, these factors have primarily been described and measured using health behavior theories such as the Theory of Planned Behavior [54] and Social Cognitive Theory [55]. Less explored in implementation research have been the psychological heuristics that shape decision making and characterize our decision-making styles and may both mediate and moderate the impact of implementation strategies. In our 3 exploratory projects, we hypothesize that psychological heuristics affect implementation strategy success. For example, risk aversion describes the human tendency to value losses more than equivalent gains [30] and may shape how a consumer responds to a financial incentive to adhere to an EBP or may make managers reluctant to *take a gamble* on innovative practices that may put quality at risk. Present bias refers to the tendency of individuals to overvalue immediate or current rewards compared with future rewards [56]. Present-biased individuals may be more responsive to financial incentives; present-biased managers may require short-term rewards that provide more immediate feedback than those commonly seen in many pay-for-performance programs that involve incentive disbursement at the end of each year. Regret aversion refers to the tendency of individuals to reduce the possibility of regret when making choices and can be deployed in the design of lotteries or other tangible or intangible rewards systems; response to such designs is likely to vary with underlying heterogeneity in regret aversion. Individuals’ sensitivity to conformity and social referents similarly can be leveraged both through descriptive comparisons (eg, “this is how you are using EBP compared with your peers”) and injunctive comparisons (eg, “these are your supervisors’ expectations of how you will use EBP”) [57]. An individual’s unique pattern of cognitive biases and decision-making styles can be described as their *behavioral phenotype* [42] and may explain individual variation in responses to implementation strategies.

Implementation science frameworks also posit the importance of organizational factors in explaining implementation success, emphasizing constructs such as organizational culture (the collective sense of how work is done in an organization), organizational climate (the collective sense of how the work environment affects psychological well-being), implementation climate (group perspective on whether use of an innovation is expected, supported, and rewarded), and implementation leadership (group beliefs about how capably a leader supports EBP implementation) [58-62]. These organizational constructs explain much of the variance in implementation outcomes [23]. They are generally measured by aggregating individual responses within organizations or organizational units following a demonstration of construct validity at the organization level. In addition to defining individual decision-making styles, behavioral phenotypes may be important organizational descriptors, which would require aggregating responses across individuals. As an exploratory objective, we will examine the validity and utility of aggregating individual behavioral phenotypes to the organizational level [63]. In other words, do people with similar behavioral phenotypes cluster in organizations, and do these aggregated phenotypes predict important implementation outcomes? Individual behavioral phenotypes could cluster within organizations if, for example, leaders only hire individuals whose psychological heuristics are consistent with their own or if the organizational culture changes employees’ heuristics. If individual behavioral phenotypes translate into an organizational construct, we will also explore questions related to organizational composition, such as how clinicians’ behavioral phenotypes compare with those of their administrators and which is more predictive of implementation and clinical outcomes.

We will refine existing, validated measures from the behavioral economics literature for assessing psychological heuristics. Once measured in patients, providers, and administrators, these phenotypes can be evaluated as mediators and moderators of implementation strategy effects.

#### Statistical Methods Incubator: Develop Novel Approaches to Test Mediating and Moderating Effects of Mechanisms

Mechanisms refer to *processes that are responsible for change* [64]; they can be considered the *active ingredients* that explain the specific ways in which implementation strategies affect implementation and client outcomes. The identification of mechanisms can lead to more efficient and tailored strategies based on the EBP of interest and the context in which it is implemented. The goals of the statistical methods incubator are to develop methods that quantify the magnitude and statistical significance of cross-level indirect effects in mediation models (the approach needed to test mechanisms) that span patient, clinician, and organizational levels and develop methods and guidelines for designing studies that have adequate statistical power to detect mediation effects in these multilevel trials. These methods are necessary to rigorously test the implementation strategies that are developed through our exploratory projects and pilot studies.

Although some research has begun to identify challenges and propose solutions for addressing mediation in simple 2-level mediation models (patients nested within providers) [65], little is known about the extension of these methods to 3-level models (patients within providers within organizations) or models that incorporate multiple measures over time. For example, questions remain about the extent to which various model specifications result in biased parameter estimates and the most effective strategies for overcoming these biases to obtain accurate parameter estimates and correct tests of statistical significance for indirect effects in 3-level models. Complications arise in these models because of the interdependence of observations within levels and because the relationships among lower-level variables can vary at different levels [65-67]. Although significant progress has been made in accounting for these design features when modeling direct effects, the modeling of indirect effects is more complicated and has not been examined with as much rigor. This deficit is particularly important in implementation studies of organizations where there can be considerable homogeneity within levels, and interventions at 1 level can have substantial effects on other levels—all of which complicates modeling [23,40,68].

There are no good guidelines to help investigators design multilevel studies so that they have adequate power to detect indirect effects of clinical interest. The importance of designing studies with adequate statistical power to detect meaningful effects is well understood [69] and accepted. Several resources are available to support researchers in ensuring that studies are adequately powered to detect main effects of interventions in both single-level trials and multilevel trials [70-72]. As the field moves to an experimental approach that requires testing of the mediating mechanisms that link implementation strategies to outcomes at multiple levels, we will have to examine indirect effects in studies that have sufficient statistical power to detect these effects, should they be present. Although methods are available to calculate statistical power for main effects in clustered trials, we know of no validated methods to calculate statistical power to detect cross-level indirect effects in multilevel trials. Without this information, investigators are unable to plan studies that are adequately powered to address questions of mechanisms. This work will build on and extend 2 approaches to test indirect effects in multilevel models—multilevel structural equation modeling [73] and the *centered within context with means reintroduced* approach [65]—to address 3-level models with mediators, interventions, and randomization at different levels. This work will result in generalizable instructions and guidelines on how to conduct multilevel mediation analysis in 3-level mediation models applicable to studying mechanisms in a wide range of implementation trials and empirical evidence supporting the need for these approaches to increase the precision and accuracy of indirect effect estimates.

### Exploratory Projects

#### Project 1: Assisting Depressed Adults in Primary Care Treatment (Patient-Level)

Improving the management of adult depression is one of the great challenges facing outpatient mental health care. As continuous antidepressant treatment tends to improve symptoms of depression [74-76], quality of life [74], and social functioning [77] as well as reduce health care costs [78], it is a cornerstone of evidence-based treatment for adult depression. Yet adults who initiate antidepressants for depression often discontinue within the first few weeks of treatment, before their medication becomes fully effective [79]. Although patient-level strategies have been highlighted in several implementation frameworks, there has been little empirical study relating to patient-level uptake of EBP [80]. We will conduct a pilot study to test whether modest time-limited escalating or de-escalating daily financial rewards for patient antidepressant use, based on behavioral economic theory, improves medication adherence and clinical outcomes of adults initiating treatment of depression. This will make a contribution to implementation science by elucidating how patient-facing implementation strategies can be used to increase the manner in which patients engage with EBP.

Tangible financial patient rewards have successfully increased a wide range of health behaviors [31,34,81-84], including medication adherence [85-88]. Financial rewards for medication adherence tend to have their strongest effects when they are provided frequently and close in time to when the medication is taken [88]. In depression, in contrast to many other conditions, it may be necessary to provide financial incentives for antidepressant adherence only during the initial weeks of treatment, when untreated depression makes nonadherence risk greatest and before the patient’s mood begins to improve [89,90]. After this point, antidepressants may help lift the patient’s mood, providing feedback to become self-reinforcing and facilitate better adherence.

Behavioral economics research has highlighted that the design and delivery of financial incentives significantly influence effectiveness [91,92]. Antidepressant therapy requires continuous medication adherence. However, the therapy’s mechanism of action makes early adherence difficult: the rewards of antidepressants (decreased depressive symptoms) do not materialize instantaneously but only after several weeks of use, whereas the costs (inconvenience and side effects) accrue in the present. With this in mind, it is possible that providing larger initial incentives that fade over time may help people to overcome initial inertia and get started. It is also possible, however, that an increasing daily incentive, which people generally prefer [93], may better leverage key behavioral principles, including the use of reference points (people compare with what they have received previously, and the increasing rewards will be thus viewed positively) and loss aversion as patients who initiate treatment face an ever-greater lost opportunity if they discontinue medications as rewards increase [94]. However, it is also possible that an increasing daily incentive, which people generally prefer [93], may better leverage key behavioral principles, including the endowment effect (ascribing more value to things because one owns them) and loss aversion as patients who initiate treatment face an ever-greater lost opportunity if they discontinue medications as rewards increase [94].

We will compare the effects of usual care, escalating, and de-escalating patient financial incentives on daily antidepressant medication adherence and depression symptom control of nonelderly adults with clinical depression (see Table 2). A three-arm pilot study will randomize 120 adults in outpatient treatment who are starting antidepressants for depression to receive either (1) usual care (n=40), (2) usual care and escalating daily financial incentives (n=40), or (3) usual care and de-escalating daily financial incentives (n=40). Participants assigned to the escalating incentive arm will receive US $2 per day, increasing to US $7 per day with daily feedback tied to use of Adheretech wireless medication devices using the Way to Health platform for the first 6 weeks of antidepressant adherence (US $189 maximum). Those assigned to the de-escalating incentive arm will receive an incentive that decreases from US $7 per day to US $2 per day for daily antidepressant adherence over the 6-week period (US $189 maximum). The study will achieve the following specific aims: (1) compare the effectiveness of usual care, escalating incentives, and de-escalating incentives on improving adherence to antidepressant therapy and reducing depressive symptoms 6 weeks following treatment initiation; (2) determine whether 6 week escalating or de-escalating financial incentives continue to improve antidepressant adherence and depressive symptom control over the 6- to 12-week period following termination of the incentives; and (3) assess the similarity of the study groups with respect to potential negative effects of incentives including regret over study participation. We will also explore potentially moderating effects of 2 psychological biases (present bias and information avoidance) on the effectiveness of the interventions to improve daily antidepressant adherence. Habit formation and decision regret will be evaluated as secondary outcomes. Given that this study is one of the first of its kind to explore financial incentives for medication adherence in individuals with psychiatric disorders, we have paid careful attention to potential ethical concerns [95]. It is of note that we will not prescribe any medication, and we leave the assessment of benefits relative to risks for each patient to that patient’s clinician. We have included the Decision Regret Scale [96], which we will monitor regularly to identify potential issues of dissatisfaction with study participation. We also plan to conduct qualitative interviews with participants asking specifically about their perspectives on financial incentives and adherence to antidepressants.

#### Project 2: App for Strengthening Services in Specialized Therapeutic Support (Clinician-Level)

Elementary school children with autism often need intensive support throughout the day [97]. Concerns about safety, behavioral challenges, and the need for a highly structured environment have resulted in an increased use of one-to-one aides at home, school, and in the community [98,99]. These aides, referred to as therapeutic support staff (TSS) in Philadelphia, usually have a bachelor’s or associate’s degree and receive limited training and supervision due to the community-based nature of their work, which often requires them to work in settings independent from their supervisors and peers [98,100]. Ideally, aides would use evidence-based interventions in the family of applied behavior analysis to help children reduce problem behaviors and increase adaptive behaviors [101,102].

Philadelphia’s Medicaid system spends more than US $80 million a year on TSS, about a third of the children’s mental health services budget. Although children with autism comprise 7% of all children served through this system, they account for 40% of TSS services. Administrators, advocates, and parents have decried the poor or unknown quality of care and outcomes associated with it, yet the very nature of the work they do makes it difficult to monitor. Our observations of TSS in the community [98], combined with our interviews with administrators and clinicians, suggest that TSS are not supported in providing high-quality, evidence-based care, in large part because of the isolating nature of their work and limited opportunity for measurement of performance, acknowledgment, and feedback.

**Table 2:** Assisting Depressed Adults in Primary care Treatment (ADAPT) Standard Protocol Items: Recommendations for Interventional Trials (SPIRIT) flow diagram.

Stages and time point			Study period					
			Screening		Baseline	Intervention period (weeks 1-6)	Follow-up	
							End of week 6	End of week 12
**Enrollment**								
	Eligibility screen	X^a^		X		—^b^	—	—
	Verbal informed consent	X		—		—	—	—
	Randomization	—		X		—	—	—
**Interventions**								
	Escalating financial incentives	—			—	X	—	—
	De-escalating financial incentives	—			—	X	—	—
	Control	—			—	X	—	—
**Assessments**								
	Patient Health Questionnaire-9	X			—	—	X	X
	Generalized Anxiety Disorder-7	—			X	—	X	X
	Beck Hopelessness Scale	—			X	—	X	X
	Theory of Planned Behavior	—			X	—	—	—
	Information avoidance	—			X	—	—	—
	Present bias	—			X	—	—	—
	Decision Regret Scale	—			—	—	X	X
	Habit formation	—			—	—	X	X
	Daily antidepressant adherence (automated collection via electronic pill bottle)	—			—	X	X	X
	Antidepressant prescription (via electronic health record abstraction)	—			—	X	X	X

^a^X denotes the study period in which each component occurs.

^b^Not applicable.

This project will develop and test strategies to increase TSS’s self-efficacy, supervision, and sense of belonging to a professional community, with opportunities for peer comparison and supervisor recognition as mechanisms to increase the use of EBP. The target practice for this study is data collection. We chose data collection because (1) it is a component of every EBP for children with autism and is common to many mental health interventions for other children as well, (2) this foundational practice is essential to measuring and monitoring outcomes and has been associated with more positive outcomes in and of itself, and (3) it makes possible the objective assessment of the effectiveness of implementation strategies and supports iteration and improvement.

Our clinician-focused implementation strategy is based on 3 psychological principles informed by behavioral economics. The first is bounded rationality, defined as limited information, cognitive capacity, and willpower [103]. TSS may find data collection difficult because they are not sure what information to collect or how to collect it easily. The second is perceptions of social norms [57]. On the basis of typical practice, TSS may believe that their supervisors do not expect them to collect data and that none of their peers collect data. The third is hyperbolic discounting, in which people prefer more immediate gratification (not expending effort to collect data) at the expense of long-term outcomes (data ultimately used to show a child’s progress and inform future interventions) [56].

Philadelphia’s Department of Behavioral Health is making substantial investments in establishing and enforcing standards for autism intervention. Data collection will be an important part of these new standards. We will use a participatory design approach to build a digital app on the Penn Way to Health platform that allows TSS to (1) receive frequent communication and reminders about how and when to collect data, (2) easily collect and upload data, (3) observe how the child in their care is progressing, (4) observe how they compare with their peers in data collection, and (5) receive positive recognition from their supervisors and employers in response to frequent and accurate data collection.

We will use rapid-cycle prototyping, an iterative development process that involves multiple tests of our intervention’s utility, feasibility, acceptability, and potential for long-term system-wide implementation. To develop the components of this new tool, we will (1) conduct an innovation tournament among TSS and their supervisors to identify ways to increase TSS’s data collection; (2) observe and query 10 TSS workers in the field to examine how they collect data, the functional and structural barriers that impede their ability and willingness to collect data, and their intentions, attitudes, subjective norms, and self-efficacy regarding data collection; (3) use rapid prototyping and testing to create an app through Way to Health that makes data collection easier, more rewarding, and socially desirable and refine the app based on observation and data collection; (4) test the refined app with 30 TSS from 3 agencies; and (5) explore broader applicability of this technology with our partners to determine how use of other EBPs can be objectively and inexpensively measured and rewarded.

#### Project 3: Motivating Outpatient Therapists to Implement: Valuing a Team Effort (Organizational-Level)

In project MOTIVATE, we will partner with stakeholders to develop implementation strategies that target organizations and leverage established principles from behavioral economics to improve EBP implementation in community mental health clinics. In our work investigating the implementation of EBP over the past 5 years [23,104], agency administrators and policy makers have repeatedly told us that the most significant barrier to implementing EBP is the need for a significant organizational financial investment, which is challenging in the context of an under-resourced public mental health system [21,105-108].

In response to this challenge, payers, including Philadelphia’s Department of Behavioral Health and Intellectual disAbility Services (DBHIDS), are beginning to use financial incentives to motivate organizations to encourage EBP implementation. These efforts are based in part on evidence from a few published studies, which show that pay-for-performance schemes, that is, paying clinicians directly for the implementation of EBPs, result in greater use of EBP and higher fidelity [109-111]. However, studies have also shown that incenting organizations rather than clinicians is not highly effective in changing clinicians’ use of EBP [112-115]. This discrepancy highlights the importance of understanding how to help organizations leverage incentives to change clinician behavior most efficiently and effectively.

The limited impact of paying organizations to change clinician behavior may be due to a number of factors ranging from poor incentive design to organizational incentives not being translated into incentives for frontline clinicians [116]. Organizational leaders likely do not have the training in how to design or use flow-through incentive funds effectively nor is there any research (or established implementation strategies) to guide this practice [115]. We address this gap through a participatory design process that integrates stakeholder input from clinicians, administrators, policy makers, and payers to develop incentive-oriented implementation strategies that target organizations [36].

Our participatory design approach incorporates 3 novel and promising methods for systematically eliciting and leveraging end-user input to design effective interventions. First, we will use an innovation tournament (described previously) to generate ideas from clinicians (the end users) about the best ways for organizations to use financial and nonfinancial incentives to facilitate clinician implementation of EBP (see description above). Second, we will refine the ideas generated through the innovation tournament using a *behavioral diagnosis* process. We will use a structured tool developed by ideas42 to comprehensively analyze the ideas submitted in the tournament and identify specific behavioral barriers impeding the use of EBPs by clinicians. For example, tournament ideas that indicate that clinicians run out of time to implement EBPs during the standard 50-min therapy session may suggest that the planning fallacy (ie, the tendency to consistently underestimate the time needed to complete an action) contributes to incomplete or infrequent use of EBPs in session. The behavioral diagnosis step will yield multiple hypothesized behavioral bottlenecks that, once confirmed or disconfirmed by stakeholders, will inform implementation strategy design.

Third, once a set of implementation strategies have been identified as potentially useful, we will use a discrete choice experiment to systematically elicit and quantify stakeholders’ preferences regarding how these strategies should be designed and structured [53]. Discrete choice experiments present potential users of a product or service with a series of forced-choice questions that require them to choose between alternative designs. For example, clinicians might be required to choose between a financial incentive in the form of a large annual bonus for high EBP fidelity or a small monthly payment for fidelity or they might choose to verify fidelity by tape recording all sessions or having 1 in-session observation. By systematically combining various attributes and levels, a discrete choice experiment quantifies the extent to which specific design features are desired. This information will then be used to inform the design of an incentive strategy that targets organizations to improve clinicians’ EBP delivery.

### Declarations

#### Current Study Status

We have begun recruitment and data collection for Projects 1 and 3; data collection is ongoing for both projects. Project 2 recruitment and data collection will begin in 2019. No publications containing the results of this study have been submitted or published in any journal.

#### Ethics Approval and Consent to Participate

The institutional review boards of the University of Pennsylvania and the City of Philadelphia approved all study procedures, and all ethical guidelines were followed. All individuals interested in participating in the research described in this protocol will provide written informed consent.

### Setting

This work is occurring in close collaboration with community stakeholders vested in mental health in the City of Philadelphia. Our major partners include the DBHIDS, the School District of Philadelphia, the nonprofit organizations that serve the mental health needs of Philadelphia residents in the DBHIDS network, and the University of Pennsylvania Health System. Philadelphia is a city of 1.6 million racially and ethnically diverse communities, approximately a third of who are enrolled in Medicaid. Philadelphia manages the behavioral health care of its Medicaid enrollees through Community Behavioral Health (CBH), a not-for-profit corporation that falls within the organizational structure of DBHIDS. CBH pays for mental health and substance use care for more than 100,000 consumers annually and has more than 250 organizations in network, including approximately 50 CBH centers and other specialized mental health and substance use agencies providing outpatient services.

The School District of Philadelphia serves approximately 140,000 students educated in 214 schools annually, making it the eighth largest school district in the United States. Furthermore, three-quarters of its students are eligible for free or reduced-price lunch. About two-thirds of students are African American and 10% are Latinx, and 107 languages other than English are spoken at home by District students.

The University of Pennsylvania Health System is the largest health system in the Delaware Valley. It includes 6 hospitals and 2 regional medical centers. Last year, the health system accommodated more than 5 million outpatient visits, 135,000 adult hospital admissions, and 350,000 emergency room visits.

All projects are approved by the appropriate institutional review boards.

## Results

Enrollment for project ADAPT started in 2018; results are expected in 2020. Enrollment for project ASSISTS will begin in 2019; results are expected in 2021. Enrollment for project MOTIVATE started in 2018; results are expected in 2019. Data collection had begun for ADAPT and MOTIVATE when this protocol was submitted.

## Discussion

### Summary

Although hundreds of treatments for common psychiatric disorders have demonstrated efficacy, problems persist in optimizing their implementation in community practice [117,118]. When evidence-based interventions are adopted, they are often not implemented in the way they were designed and do not result in the same outcomes observed with highly selected patients under controlled conditions. The 3 projects described in this protocol address the problems of incomplete uptake of selected EBPs in community practice and will result in approaches that could lay the foundation for ways to address implementation gaps at the levels of individuals in treatment, clinicians, and organizations. Our Methods Core will develop statistical, participatory design, and behavioral phenotyping approaches to increase the specificity and external validity of implementation strategies and the rigor with which and for whom they work.

One innovation of our Penn ALACRITY center is the merging of mental health treatment, implementation science, behavioral economics, and participatory design. Implementation research in mental health has identified important, malleable characteristics of treatments, the individuals using them, and the organizations in which they work that affect their use and outcomes. Behavioral economists have identified a wide range of patterns in human decision making that may contribute to poor health outcomes as well as methods of designing the environment to optimize optimal decision making [91,92,119-127]. These complementary approaches may be helpful when considering the implementation challenges faced in the community. Participatory design actively involves stakeholders in designing new technologies to help ensure that the results meet their needs. Our Penn ALACRITY center activities will introduce a new level of rigor and innovative methods for eliciting and incorporating stakeholder input and will represent the first merging of interdisciplinary perspectives. Future research can test the output of these different participatory design approaches to test their effectiveness in the design of implementation strategies.

### Outcomes and Conclusions

We anticipate that our Penn ALACRITY center will result in the following outcomes. First, through the design, measurement, and statistical methods incubators, the Methods Core will generate guidelines for using participatory design approaches (ie, innovation tournaments, rapid-cycle prototyping, and discrete choice experiments) in the development of implementation strategies; a set of publicly available measures of behavioral phenotypes and a database that pools deidentified patient, provider, and organizational data; and practical statistical tools, guidelines, and information that facilitate the design of the next generation of mechanism-focused randomized trials in implementation science. Second, through our exploratory projects, we will generate data that will seed future implementation science studies that incorporate advances from behavioral economics and participatory design [128]. Third, through our scientific and dissemination activities, we hope both to (a) advance implementation science by integrating new conceptual models and methods to develop implementation strategies and (b) increase the use of EBPs in community settings to improve patient well-being and quality of care.

## Abbreviations

ADAPT: Assisting Depressed Adults in Primary care Treatment
ALACRITY: Advanced Laboratories for Accelerating the Reach and Impact of Treatments for Youth and Adults with Mental Illness
ASSISTS: App for Strengthening Services In Specialized Therapeutic Support
CBH: Community Behavioral Health
DBHIDS: Department of Behavioral Health and Intellectual disAbility Services
EBP: evidence-based practice
MOTIVATE: Motivating Outpatient Therapists to Implement: Valuing a Team Effort
TSS: therapeutic support staff
